# The effect of piston diameter in primary stapes surgery on surgical success

**DOI:** 10.1007/s00405-023-08407-w

**Published:** 2024-01-26

**Authors:** Esther E. Blijleven, Maaike Jellema, Robert J. Stokroos, Inge Wegner, Hans G. X. M. Thomeer

**Affiliations:** 1 Department of Otorhinolaryngology-Head and Neck Surgery, University Medical Center Utrecht, P.O. Box 85500, 3508 GA Utrecht, The Netherlands; 2 Brain Center, University Medical Center Utrecht, Utrecht, The Netherlands; 3 Department of Otorhinolaryngology-Head and Neck Surgery, University Medical Center Groningen, Groningen, The Netherlands

**Keywords:** Otosclerosis, Hearing loss, Stapes surgery, Pure-tone audiometry, Speech perception, Otology, Piston diameter

## Abstract

**Purpose:**

To evaluate the effect of piston diameter in patients undergoing primary stapes surgery on audiometric results and postoperative complications.

**Methods:**

A retrospective single-center cohort study was performed. Adult patients who underwent primary stapes surgery between January 2013 and April 2022 and received a 0.4-mm-diameter piston or a 0.6-mm-diameter piston were included. The primary and secondary outcomes were pre- and postoperative pure-tone audiometry, pre- and postoperative speech audiometry, postoperative complications, intraoperative anatomical difficulties, and the need for revision stapes surgery. The pure-tone audiometry included air conduction, bone conduction, and air–bone gap averaged over 0.5, 1, 2 and 3 kHz.

**Results:**

In total, 280 otosclerosis patients who underwent 321 primary stapes surgeries were included. The audiometric outcomes were significantly better in the 0.6 mm group compared to the 0.4 mm group in terms of gain in air conduction (median = 24 and 20 dB, respectively), postoperative air–bone gap (median = 7.5 and 9.4 dB, respectively), gain in air–bone gap (median = 20.0 and 18.1 dB, respectively), air–bone gap closure to 10 dB or less (75% and 59%, respectively) and 100% speech reception (median = 75 and 80 dB, respectively). We found no statistically significant difference in postoperative dizziness, postoperative complications and the need for revision stapes surgery between the 0.4 and 0.6 mm group. The incidence of anatomical difficulties was higher in the 0.4 mm group.

**Conclusion:**

The use of a 0.6-mm-diameter piston during stapes surgery seems to provide better audiometric results compared to a 0.4-mm-diameter piston, and should be the preferred piston size in otosclerosis surgery. We found no statistically significant difference in postoperative complications between the 0.4- and 0.6-mm-diameter piston. Based on the results, we recommend always using a 0.6-mm-diameter piston during primary stapes surgery unless anatomical difficulties do not allow it.

**Supplementary Information:**

The online version contains supplementary material available at 10.1007/s00405-023-08407-w.

## Introduction

Otosclerosis is characterized by abnormal bone remodeling [1]. Bone overgrowth can cause fixation of the stapes footplate, which can lead to conductive hearing loss, vertigo and/or tinnitus [2]. The hearing loss can be treated by a surgical procedure called stapes surgery. During this surgical procedure, the stapes will be partly removed and replaced by a prosthesis, also known as a piston [3]. Stapes surgery is a highly successful procedure, as 72–95% of adult patients have postoperative air–bone gap (ABG) closure to 10 dB or less [4–6].

Over the years, a large number of pistons have been developed to improve postoperative hearing results. The diameter of these pistons vary from 0.3 to 0.8 mm. Mathematical models and temporal bone studies indicate that the acoustic transmission is better if the surgeon uses a piston with a larger diameter, which will improve postoperative pure-tone audiometry (PTA) results [3, 7–11]. However, it is believed that using a smaller diameter piston reduces the risk of iatrogenic trauma and consequent sensorineural hearing loss (SNHL) [7].

Numerous studies have already evaluated the effect of piston size on postoperative hearing results, but the majority of the results were inconclusive due to several limitations [3, 11–20]. A published systematic review on the effect of piston diameter in stapes surgery on hearing results showed that the sample sizes of previous studies were relatively small. A power analysis shows that 202 patients per diameter group are needed to detect a 10% difference in surgical success between two piston diameter [7]. Moreover, not all studies evaluated postoperative ABG and success rate as recommended by the American Academy of Otolaryngology-Head and Neck Surgery Committee on Hearing and Equilibrium [21]. Lastly, anatomical difficulties that may have influenced the choice of piston diameter, were not evaluated in all studies [7, 11, 22].

A lack of sufficiently powered clinical studies on the subject demonstrates the need for the publication of our results. To accommodate this need, we evaluated the effect of piston diameter on postoperative hearing results and complications in otosclerosis patients undergoing primary stapes surgery.

## Methods

### Study design and population

A retrospective, single-center cohort study was performed in a tertiary referral center in the Netherlands. Adult otosclerosis patients who underwent primary stapes surgery between January 2013 and April 2022 and who received a 0.4- or a 0.6-mm-diameter piston were included. Surgeries were performed by five ENT surgeons with more than 5 years of experience in performing stapes surgery. Some patients underwent primary stapes surgery on both ears. Each ear was analyzed as a separate case and therefore we refer to cases instead of patients throughout this article. Cases were excluded if postoperative audiometric results were not available or if they received a 0.3- or 0.8-mm-diameter piston. Pistons with a 0.3- and 0.8-mm diameter are rarely used at our center, while both 0.4- and 0.6-mm-diameter pistons are most commonly used.

### Intervention

In all cases, an endaural procedure with or without intercartilaginous incision was performed. Rosen’s incision was used to raise the endomeatal flap and after identification of the annulus fibrosis it was lifted. The ossicles, chorda tympani nerve and the facial nerve were identified. The surgeon inspected if the stapes was sclerotic and fixed. If so, the incudostapedial joint was cleaved, the stapedius muscle and the posterior crus of the stapes were transected and the anterior crus of the stapes was breached to remove the stapes superstructure. The stapes footplate was fenestrated with a KTP laser (Lumenis, Inc., Salt Lake City, Utah, USA), a Skeeter microdrill (Medtronic Xomed Inc, Jacksonville, Florida, USA), microinstruments or a combination of these. A Causse loop Teflon piston, a Kurz titanium piston or a Teflon wire piston was placed between the incus and the fenestration in the stapes footplate. Two groups of cases were identified: a group of cases that received a 0.4-mm-diameter piston and a group of cases that received a 0.6-mm-diameter piston. Depending on possible anatomical difficulties, the exact piston diameter (0.4 or 0.6 mm) and length were determined. Blood clots and/or allogeneic tissue (Gelfoam, Pfizer, New York, New York, USA) were used to seal the oval window.

### Outcomes

The following variables were reviewed and tabulated in a computer database: age, gender, existence of bilateral otosclerosis, intraoperative anatomical difficulties, piston diameter, postoperative complications such as SNHL and vertigo, need for revision surgery and reason for revision surgery. Severe SNHL was defined as a postoperative bone conduction (BC) of > 70 dB and was only noted if the patient experienced postoperative hearing loss. Vertigo was only noted when nystagmus was objectified by medical personnel.

### Pure-tone audiometry

In all cases, the last preoperative PTA, the first postoperative PTA and the one-year postoperative PTA were used for analysis. The PTA results consisted of the air conduction (AC) and BC thresholds measured at 0.5, 1, 2 and 3 kHz, as recommended by the 1995 American Academy of Otolaryngology Head and Neck Surgery Committee on Hearing and Equilibrium guidelines [21]. In the Netherlands, thresholds at 3 kHz are not routinely measured, so we interpolated 3 kHz thresholds by averaging the thresholds at 2 and 4 kHz [22]. The pre- and postoperative AC and BC thresholds at 0.5, 1, 2 and 3 kHz and the corresponding air–bone gaps were averaged. In some cases, BC and AC thresholds at 4 kHz exceeded the maximum volume that our center’s audiometer can produce. In these cases, we could not calculate 3 kHz thresholds, because we could not detect 4 kHz thresholds. Therefore, we averaged the thresholds at 0.5, 1, and 2 kHz in 31 cases. BC and AC thresholds that were used for calculation of the ABG were obtained at the same time. ABG closure to 10 dB or less and ABG closure to 20 dB or less were calculated.

We compared the first postoperative PTA results with the one-year postoperative results, to evaluate a possible change in audiometric results over time. The PTA measurements were also evaluated with the Amsterdam Hearing Evaluation Plots [23].

### Speech audiometry

The speech discrimination score (SDS), speech reception threshold (SRT) and 100% speech reception were analyzed. The speech discrimination score is the percentage of words that a patient correctly repeats at 60 or 65 dB. The speech reception threshold is the dB level at which the patient correctly repeats 50% of the words. The 100% speech reception is the dB level at which a patient correctly repeats 100% of the words.

### Statistical analysis

Medians and interquartile ranges (IQRs) were calculated for continuous variables. Frequency and percentages were calculated for categorical variables. A test for normality, the Shapiro–Wilk test, was used to assess whether variables were normally distributed. Since all our outcomes were not-normally distributed, continuous variables were tested using the Mann–Whitney* U* test. Categorical variables were tested using Fisher’s exact test. The statistical analyses were performed using IBM SPSS Statistics version 26.0 (IBM Corp., Armonk, NY, USA).

## Results

### Study population

In total, 315 otosclerosis patients underwent 363 primary stapes surgeries. Thirty-two cases were excluded because they received a 0.3-mm-diameter piston or a 0.8-mm-diameter piston and 10 cases were excluded because no postoperative audiometric results were available. Therefore, we included a total of 280 patients who underwent 321 primary stapes surgeries. A total of 246 cases received a 0.4-mm-diameter piston and 75 cases received a 0.6-mm-diameter piston. Five cases were excluded from evaluation of the BC and ABG, because in one case no bone conduction was measured and in four cases it was likely that the postoperative BC was measured incorrectly. In these four patients the postoperative BC was unmeasurable. However, three of these patients experienced a subjective substantial improvement in their hearing and showed an improvement in AC threshold postoperatively. One patient experienced no improvement or deterioration of hearing.

Median age was 47 years (IQR 17) in the 0.4 mm group versus 48 years (IQR 13) in the 0.6 mm group (Appendix 1). In the 0.4 mm group, 67% of cases were female compared to 61% in the 0.6 mm group. Bilateral otosclerosis was present in 60% of patients in the 0.4 mm group compared to 71% in the 0.6 mm group. These differences in baseline characteristics were neither clinically relevant nor statistically significant. Anatomical difficulties were significantly more common in the 0.4 mm group (7%) than in the 0.6 mm group (0%, *p-*value 0.016). No anatomical difficulties arose in the 0.6 mm group. In the 0.4 mm group, three cases had an overhanging facial nerve, seven cases had a dehiscent facial nerve and six cases had a narrow oval window niche.

### Pure-tone audiometry

The median duration between preoperative PTA measurement and stapes surgery was 4 weeks with a range of 0–79 weeks. The median duration of first postoperative PTA was 7 weeks with a range of 1–26 weeks. The preoperative PTA results did not differ significantly between both groups (Tables 1, 2). We found postoperative improvements in the audiometric results in both groups. The gain in AC was significantly larger in the 0.6 mm group (median 24 dB, IQR 15) compared to the 0.4 mm group (median 20 dB, IQR 14). The postoperative ABG was significantly smaller in the 0.6 mm group (median 8, IQR 7) compared to 0.4 mm group (median 9 dB, IQR 7). ABG closure to 10 dB or less was significantly higher in the 0.6 mm group (75%) compared to the 0.4 mm group (59%). There was no statistically significant difference in the ABG closure to 20 dB or less between the 0.4 and 0.6 mm group.

**Table 1:** **A**ir conduction

	Piston diameter 0.4 mm (*n *= 246)	Piston diameter 0.6 mm (*n* = 75)	*p*-Value
Preoperative AC Median (IQR), dB	52.5 (16.3)	56.3 (18.8)	0.213
Postoperative AC median (IQR), dB	30.0 (15.6)	31.9 (20.6)	0.479
Gain AC Median (IQR), dB	20.0 (14.4)	24.4 (15.0)	**0.011**

*p*-Value is calculated as the difference between the two piston sizes, *p*-value < 0.05 is statistically significant and displayed in bold

*AC* air conduction; *IQR* interquartile range

**Table 2:** Bone conduction and air–bone gap

	Piston diameter 0.4 mm (*n* = 243)	Piston diameter 0.6 mm (*n* = 73)	*p*-Value
Preoperative BC Median (IQR), dB	23.8 (10.0)	26.3 (15.0)	0.068
Postoperative BC Median (IQR), dB	20.6 (14.4)	25.0 (17.2)	0.220
Gain BC Median (IQR), dB	3.1 (7.5)	3.1 (7.5)	0.608
Preoperative ABG Median (IQR), dB	27.5 (11.3)	26.3 (14.4)	0.617
Postoperative ABG Median (IQR), dB	9.4 (6.9)	7.5 (6.9)	** < 0.001**
Gain ABG Median (IQR), dB	18.1 (13.8)	20.0 (15.7)	**0.035**
ABG closure ≤ 10 dB *n* (%)	144 (59.3)	55 (75.3)	**0.013**
ABG closure ≤ 20 dB *n* (%)	230 (94.7)	72 (98.6)	0.147

*p*-Value is calculated as the difference between the two piston sizes, *p*-value < 0.05 is statistically significant and displayed in bold

*ABG* air–bone gap; *IQR* interquartile range

PTA was performed at 12-months follow-up in 91 cases, as reported in Table 3. We found no significant change in AC and ABG between first postoperative PTA and the 1-year postoperative PTA for both groups.

**Table 3:** Change in pure-tone audiometry results over time

	Number of cases, *n* 0.4/0.6 mm	Piston diameter 0.4 mm	Piston diameter 0.6 mm	*p*-Value
Change in AC Median (IQR), dB	74/14	0.0 (6.4)	1.6 (5.2)	0.576
Change in ABG Median (IQR), dB	70/14	0.0 (7.0)	−0.9 (5.2)	0.340

*p*-Value is calculated as the difference between the two piston sizes, *p*-value < 0.05 is statistically significant

The calculated change is the difference of the hearing results after ± 1 year and the first postoperative measurement after ± 6 weeks, except for one patient who had his first postoperative measurement at ± 6 months postoperatively

*AC* air conduction; *ABG* air–bone gap

**Fig. 1:**
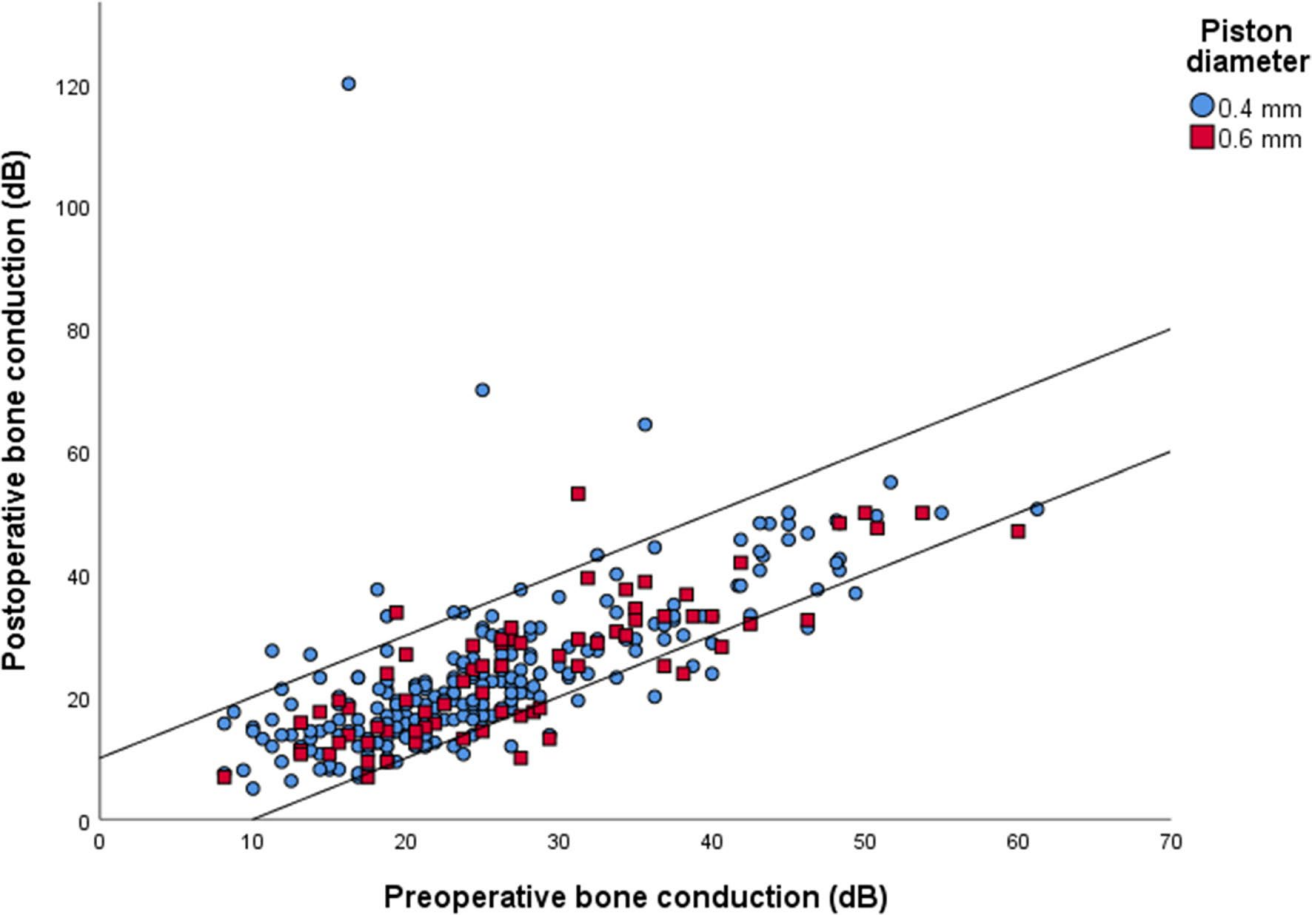
Amsterdam hearing evaluation plot (*n* = 321). The cases above the upper diagonal line have a postoperative bone conduction (BC) loss of more than 10 dB, which may be a sign of cochlear damage. In the cases enclosed by the two diagonal lines, BC thresholds were not affected more than 10 dB. The cases below the lower diagonal line present cases that experienced an improvement of the BC of more than 10 dB, most likely due to the Carhart effect

**Fig. 2:**
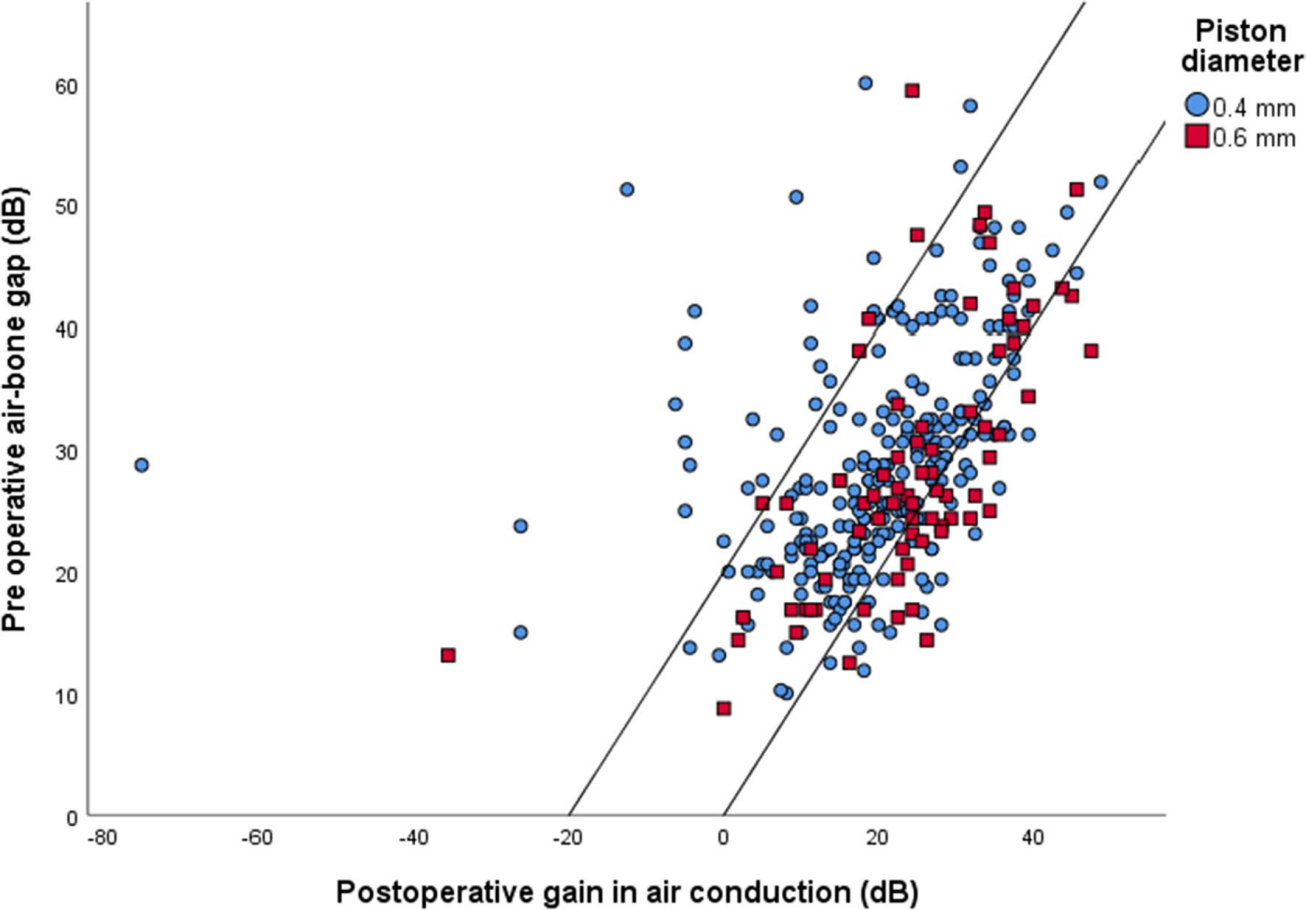
Amsterdam hearing evaluation plot (*n* = 321). The cases enclosed by the two diagonal lines have a favorable outcome. In these patients, the air–bone gap (ABG) was closed to 20 dB or less. All cases below the lower diagonal line are defined as overclosure and these surgeries are also considered as stapes surgeries with ABG closure to 10 dB or less. In all cases above the upper diagonal line no ABG closure of 20 dB or less was achieved

Figure 1 shows the postoperative change in BC stratified by piston diameter. Nine cases (4%) in the 0.4 mm group and two cases (3%) in the 0.6 mm group developed BC loss of more than 10 dB. One case (0.4%) in the 0.4 mm group developed a deaf ear. Figure 2 shows ABG closure to 20 dB or less stratified by piston diameter. ABG closure to 20 dB or less was achieved in 230 cases (95%) in the 0.4 mm group and in 72 cases (99%) of the 0.6 mm group.

### Speech audiometry

Table 4 shows the speech reception thresholds, speech discrimination scores and 100% speech receptions stratified by piston diameter. We found no statistically significant differences in gain of speech reception threshold and gain in speech discrimination score between the 0.4 and 0.6 mm group. The 100% speech reception was significantly lower in the 0.4 mm group (median 80 dB, IQR 20) than in the 0.6 mm group (median 75 dB, IQR 25).

**Table 4:** Speech discrimination and speech reception score

	Piston diameter 0.4 mm (*n* = 203)	Piston diameter 0.6 mm (*n* = 66)	*p*-Value
Preoperative SRT Median (IQR), dB	74.0 (18.0)	74.5 (19.0)	0.523
Postoperative SRT Median (IQR), dB	53.0 (14.0)	51.5 (17.0)	0.207
Gain SRT Median (IQR), dB	19.0 (16.0)	23.0 (19.0)	0.052
Preoperative SDS Median (IQR), %	0.0 (30.0)	0.0 (32.0)	0.712
Postoperative SDS Median (IQR), %	81.0 (46.0)	85.0 (61.0)	0.107
Gain SDS Median (IQR), %	58.0 (64.0)	61.5 (75.5)	0.472
100% speech reception Median, dB	80.0 (20.0)	75.0 (25.0)	**0.034**

p-Value is calculated as the difference between the two piston sizes, *p*-value < 0.05 is statistically significant and displayed in bold

*SRT* speech reception threshold; *SDS* speech discrimination score; *SD* standard deviation

### Complications and revision stapes surgery

Table 5 presents the incidence of postoperative dizziness, postoperative complications, revision stapes surgery and primary cause of failure. We found no statistically significant difference in incidence of revision stapes surgery, primary cause of failure and postoperative complications between both groups. One case (0.4%) in the 0.4 mm group had severe postoperative SNHL. The incidence of the complication vertigo was similar in both groups. The main indication for revision stapes surgery was a short piston.

**Table 5:** Postoperative dizziness, complications and revision

	Group 1 (0.4 mm)	Group 2 (0.6 mm)	Total	*p*-Value
Number of cases, *n*	246	75	321	
Postoperative dizziness, *n* (%)	55 (22.4)	22 (29.3)	77 (24.0)	0.220
Revision, *n* (%)	16 (6.5)	2 (2.7)	18 (5.6)	0.262
Cause of revision, *n* (%)				0.359
Malleus fixation	2 (0.8)	0 (0)	2 (0.6)	
Incus erosion	2 (0.8)	0 (0)	2 (0.6)	
Piston too short	7 (2.8)	0 (0)	7 (2.2)	
Piston too long	1 (0.4)	1 (1.3)	2 (0.6)	
Dislocation	1 (0.4)	1 (1.3)	2 (0.6)	
Perilymph leakage	2 (0.8	0 (0)	2 (0.6)	
Missing	1 (0.4)	0 (0)	1 (0.3)	
Complications, *n* (%)	13 (5.3)	3 (4.0)	16 (5.0)	1.000
Specification complications *n* (%)				
Vertigo	3 (1.2)	1 (1.3)	4 (1.2)	
Tinnitus	5 (2.0)	0 (0)	5 (1.5)	
Severe SNHL	1 (0.4)	0 (0)	1 (0.3)	
Prolonged hospitalization	1 (0.4)	0 (0)	1 (0.3)	

Severe SNHL is defined as an postoperative bone conduction of > 70 dB; Prolonged hospitalization is a duration of stay > 48 h

*n.a*. non applicable

## Discussion

### Summary of main results

In this study, we included 321 cases to evaluate the effect of piston size on audiometric results and postoperative complications. In both groups we found significant improvements in the postoperative audiometric results. The audiometric outcomes were significantly better in the 0.6-mm-diameter group compared to the 0.4-mm-diameter group in terms of gain in air conduction (median 24 and 20 dB, respectively), postoperative air–bone gap (median 8 and 9 dB, respectively), air–bone gap closure to 10 dB or less (75% and 59%, respectively) and 100% speech reception (median 75 and 80 dB, respectively). Although the mean postoperative air bone gap difference between the 0.4-mm-diameter group and 0.6-mm-diameter group was statistically significant. We do not consider the 1 dB to be clinically relevant. We found no higher incidence of revision stapes surgeries or postoperative complications in the 0.6-mm-diameter group compared to the 0.4-mm-diameter group. Anatomical difficulties were more common in the 0.4-mm-diameter group (7% in the 0.4 mm group compared to none in the 0.6 mm group).

### Literature overview

Table 6 compares our results with previously published results on the effect of piston diameter on audiometric results [12, 14, 17–20, 24–26]. Most of the previously published studies showed no significant difference between piston sizes and the results of these studies were not consistently in favor of a 0.4- or a 0.6-mm-diameter piston. Four studies found a significant difference in audiometric results in favor of the 0.6-mm-diameter piston. Bernardeshi et al. and Forton et al. found a significantly better gain in AC and Faranaesh et al. and Casale et al. found a significantly better gain in BC [18, 24]. However, Rompaey et al. showed more cases with ABG closure to 10 dB or less in the 0.3- and 0.5-mm-diameter groups (56% and 60%, respectively) than in the 0.4- and 0.6-mm-diameter groups (32% and 33%, respectively) [17]. The sample sizes of all included studies were smaller than our sample size, but the sample size of the 0.6-mm-diameter group of Rompaey et al. was larger compared to our group size (*n *= 105 and 75, respectively) [17].

**Table 6:** Comparison of literature

Study	No. of cases	Air conduction			Bone conduction			Air–bone gap closure	
		Preoperative (dB)	Postoperative (dB)	Gain (dB)	Preoperative (dB)	Postoperative (dB)	Gain (dB)	≤ 10 dB (%)	≤ 20 dB (%)
Current study (median)									
0.4 mm	248	53	30	**20**	24	21	3	**59**	**94**
0.6 mm	75	56	32	**24**	26	25	3	**73**	**96**
Salvador [20]									
0.4 mm	50	55	35	21	26	24	2.1*	62	n/a
0.6 mm	75	54	32	22	25	22	3.3*	71	
Bernardeschi [18]									
0.4 mm	50	48	27	**20**	25	21	4*	90	n/a
0.6 mm	50	49	25	**24**	26	19	6	94	
Shah [19]									
0.4 mm	10	60	32*	27	19	23	−4*	n/a	n/a
0.6 mm	4	59	33*	26	25	22	3*		
Faranesh [24]									
0.4 mm	9	53	26	27	16	16	−**2**	56	n/a
0.6 mm	9	55	22	32	18	12	**4**	56	
Van Rompaey [17]									
0.4 mm	155	n/a	n/a	n/a	n/a	n/a	n/a	32	96
0.6 mm	105							33	99
Cotulbea [14]									
0.4 mm	207	n/a	n/a	n/a	n/a	n/a	n/a	64	90
0.6 mm	49							63	88
Forton [25]									
0.4 mm	34	n/a	n/a	**25**	n/a	n/a	n/a	n/a	n/a
0.6 mm	28			**31**					
Casale [12]									
0.4 mm	30	n/a	n/a	n/a	n/a	n/a	**7**	27	93
0.6 mm	30						**9**	27	93
Mangham [26]									
0.4 mm	13	n/a	n/a	n/a	n/a	n/a	n/a	69	95
0.6 mm	75							92	97

*n/a* not available

*p*-Value < 0.05 is statistically significant and displayed in bold

*Calculated

The published literature states that audiometric results improve over time and that optimal hearing results take longer to achieve when using a smaller piston diameter [27]. Patients who received a 0.4-mm-diameter piston achieved optimal audiometric results at a later time than the patients who received a 0.6-mm-diameter piston. We were unable to compare the audiometric results of all included cases over time, as patients usually undergo audiometry at 6- to 8-weeks follow-up only in our center. In 91 cases, we found no significant differences in PTA results over time, but the risk of bias may be high due to selection bias. In particular, patients with persistent hearing loss may be more inclined to visit the ENT outpatient clinic 1 year postoperatively for a PTA, so these 91 cases may not be a representative sample for the study population as a whole. However, patients who have undergone middle ear surgery on the other ear will also visit the ENT outpatient clinic one year postoperatively. In these bilateral cases, the contralateral ear is also tested during the preoperative visit, providing PTA results from the contralateral ear 1 year postoperatively.

### Postoperative complications

Only one patient in the 0.4 mm group developed severe SNHL due to surgery. No adverse event occurred (such as a Gusher, labyrinthitis or malposition of the prosthesis) during the operative procedure or in the postoperative course. It remains elusive why this patient lost his hearing. This indicates that the placement of the larger piston diameter does not appear to result in additional risk of iatrogenic cochlear damage, which is in line with previous findings [12, 14, 18–20, 25, 26].

The study of Gupta et al. found a higher incidence of postoperative vertigo in patients who received a 0.6 mm piston size [16]. However, no actual numbers were reported in their article. In our study, the incidence of vertigo was relatively low (1% in both groups). The incidence of dizziness was higher in both groups, namely 22% in the 0.4-mm-diameter group and 29% in the 0.6-mm-diameter group. In most cases, these symptoms disappeared after a few days. Transient dizziness could be considered as a normal reaction of the vulnerable labyrinth after inner ear surgery.

### Patient-reported outcome measures

Several included cases showed discrepancies between the objective PTA measurements and the degree of patient satisfaction. For example, in some cases, patients experienced a substantial improvement in their hearing, while the ABG was not closed to 20 dB or less. It might be valuable to consider a patient’s perception of their postoperative hearing when evaluating the effect of stapes surgery instead of the audiometric results alone. Patient-reported outcome measures are questionnaires that can be used to assess a patient’s perception of their health status and quality of life. The Stapesplasty Outcome Test (SPOT-25) is a patient-reported outcome measure and is currently being validated in Dutch otosclerosis patients. In the future, our center will be able to measure surgical success in the Netherlands from the patient perspective [28].

### Strengths and limitations

Our study had different strengths. First of all, baseline characteristics, including preoperative audiometric results, were similar in both study groups. Secondly, we were able to include a large sample size in comparison to previous published studies. When it comes to limitations, this is a retrospective study and therefore patients were not randomized. Due to lack of randomization there might be confounding by indication, as the surgeon makes a final decision about the piston size during surgery resulting in selection bias. In case of anatomical difficulties, for example a small window niche, the ENT surgeon always choses a 0.4-mm-diameter piston instead of a 0.6-mm-diameter piston. Using a smaller piston size allows the ENT surgeon to easily see around the piston, resulting in better visualization of the stapes footplate. However, anatomical difficulties were only described in the surgery report in 5% of all cases [11]. Using a smaller piston size was mostly a choice of habit and not surgeon-dependent surgeon’s preference. In the past, the 0.4 mm was used more often in our center, while the 0.6 mm has been used more often in recent years following a systematic review of the literature performed by our research group [7].

## Conclusion

The use of a 0.6-mm-diameter piston during primary stapes surgery seems to lead to better audiometric results compared to the use of a 0.4-mm-diameter piston. We found no statistically significant difference in postoperative complications between the 0.4 and 0.6-mm-diameter piston. Based on the results, we recommend using a 0.6-mm-diameter piston during primary stapes surgery unless anatomical difficulties do not allow it.

### Supplementary Information

Below is the link to the electronic supplementary material.

Supplementary file1 (DOCX 16 KB)

## Data Availability

Data is available upon reasonable request.
