# Supplementary material for: The effect of piston diameter in primary stapes surgery on surgical success
Source: Eur Arch Otorhinolaryngol. 2024 Jan 26;281(6):2931–9. doi: 10.1007/s00405-023-08407-w (PMC11065942; doi:10.1007/s00405-023-08407-w)
Supplement: Supplementary file 1 — Supplementary file1 (DOCX 16 KB) [file 405_2023_8407_MOESM1_ESM.docx]

**The effect of piston diameter in primary stapes surgery on surgical success**

Esther E. Blijleven MD^1,2^, Maaike Jellema, MD^1,2^, Robert J. Stokroos MD^1,2^, PhD, Inge Wegner MD^3^, PhD, Henricus G.X.M. Thomeer MD^1,2^, PhD

1. Department of Otorhinolaryngology – Head and Neck Surgery, University Medical Center Utrecht, Utrecht, the Netherlands
2. Brain Center, University Medical Center Utrecht, Utrecht, the Netherlands
3. Department of Otorhinolaryngology – Head and Neck Surgery, University Medical Center Groningen, Groningen, the Netherlands

**Corresponding author:**e.e.blijleven-2@umcutrecht.nl

**Table Baseline characteristics**

| **Characteristics**  Number of cases, n | **Group 1** (0.4 mm)   246 | **Group 2** (0.6 mm)   75 | **Total**   321 | **P-value** |
| --- | --- | --- | --- | --- |
| Median age, y (IQR) | 47.0 (17.0) | 48.0  (13.0) | 47.0  (15.0) | 0.535 |
| Gender  Female, n (%)  Male, n (%) | 165 (67.1) 85 (32.9) | 46 (61.3) 29 (38.7) | 211 (65.7) 110 (34.3) | 0.405 |
| Bilateral otosclerosis, n (%) | 148 (60.2) | 53 (70.7) | 201 (62.6) | 0.104 |
| Anatomical difficulties, n (%) | 17 (6.9) | 0 (0) | 17 (5.3) | **0.016** |
| Specification anatomical difficulties, n (%)  Overhanging facial nerve  Dehiscent facial nerve  Narrow oval window niche | 3 (1.2)  7 (2.8)  6 (2.4) | 0 (0)  0 (0)   0 (0) | 3 (0.9)  7 (2.2)  6 (1.9) | 0.233 |

IQR – interquartile range; P-value is calculated as the difference between the two piston sizes, p-value < 0.05 is statistically significant and displayed in bold; Complications – the number of cases that developed one or more complications;
